# Miniplates and mini-implants: bone remodeling as their biological foundation^1^

**DOI:** 10.1590/2177-6709.20.6.016-031.oin

**Published:** 2015

**Authors:** Alberto Consolaro

**Affiliations:** 2 Full professor, University of São Paulo (USP), School of Dentistry, Bauru, São Paulo, Brazil and University of São Paulo (USP), School of Dentistry, Ribeirão Preto, São Paulo Brazil

**Keywords:** Bone remodeling, Miniplates, Mini-implants, Dental implants, Orthodontic movement

## Abstract

The tridimensional network formed by osteocytes controls bone design by coordinating cell activity on trabecular and cortical bone surfaces, especially osteoblasts and clasts. Miniplates and mini-implants provide anchorage, allowing all other orthodontic and orthopedic components, albeit afar, to deform and stimulate the network of osteocytes to command bone design remodeling upon "functional demand" established by force and its vectors. By means of transmission of forces, whether near or distant, based on anchorage provided by miniplates, it is possible to change the position, shape and size as well as the relationship established between the bones of the jaws. Understanding bone biology and the continuous remodeling of the skeleton allows the clinician to perform safe and accurate rehabilitation treatment of patients, thus increasing the possibilities and types of intervention procedures to be applied in order to restore patient's esthetics and function.

## Resumo

A rede tridimensional formada pelos osteócitos controla o *design*ósseo ao coordenar a atividade das células nas superfícies trabeculares e corticais, especialmente os osteoblastos e os clastos. Miniplacas e mini-implantes servem de ancoragem para que os demais componentes ortodônticos e ortopédicos possam - mesmo que à distância - deformar e estimular a rede de osteócitos a comandar uma remodelação no *design* de acordo com a "demanda funcional" criada pela forças aplicadas e seus vetores. Na transmissão próxima ou longínqua de forças, com base na ancoragem oferecida pela miniplacas, pode-se mudar a posição, o formato, tamanho e relação entre os ossos maxilares. Compreender a biologia óssea e a remodelação constante do esqueleto permite atuar com segurança e precisão nesse tipo de planejamento reabilitador dos pacientes, aumentando as possibilidades e tipos de intervenção para devolver a estética e função a eles.

Bone growth physically shapes us until adulthood, approximately until 22 to 24 years old. Continuous cell proliferation and deposition of bone matrix take turns with resorption sites and moments, which allows us to claim that, during growth, bone modeling also comprises remodeling phenomena. Changes in shape resulting from growth are associated with bone remodeling for necessary functional adaptation. After this phase, the bones keep on changing every day, so as to adapt to functional demands imposed by human activity. This dynamics and the process of ongoing adaptation are what characterize bone remodeling or bone turnover.

The human skeleton gives support to soft tissues, enclosing and protecting our organs. The human skeleton is composed of 206 bones of flawless individual design that allows it to meet functional demands, in addition to absorbing, producing or transferring force during movements performed by the body. A newborn might have up to 300 bones, since a number of bones fuse during childhood and puberty.

In an embryo, each part of the mesenchyme, from which a group of cells will give rise to osteoblasts and chondroblasts, has a site of bone growth. Cell movement and continuous deposition of tissue matrix at this site imply the production of forces that add up or act in opposite directions, thereby resulting in predominant forces also known as vectors or, to be more specific, bone growth vectors. The mesenchyme is the primary tissue that will give rise to bone and cartilage, in nuclei or cores, which later on might fuse or remain independent from each other, with the formation of sutures filled by fibrous connective tissue.

## Tensegrity and mechanotransduction: the language of stimuli that sculpture the bone!

In the body of animals and vegetables, as well as in objects, natural supporting systems tend to receive and produce forces throughout their structures. However, in the end, those forces neutralize each other with a resultant force that equals to zero. Once the action of those forces, whether originated internal or externally, cease with a resultant force that equals to zero, the object or anatomical body part will remain as it were in the first place. This signals to a complete and perfect system of force distribution, a balance, of which property is named tensegrity. It applies to an overpass, a palace, the head and other members of the body, for example; but it also applies to the simplest of things, such as an earthen jar and teeth stably positioned at the dental arch. Every structure returning to its original shape upon the application of force is under structural and functional balance; however, should it have its shape modified by force and be unable to adapt to the new situation (with new tensegrity), it is natural that it tends to go back to its original shape, so as to establish balance.

In a set of muscles and tendons, and/or interconnected bones, every time tensegrity between a cell and its cytoskeleton is broken, cell components will release a number of mediators with a view to restoring its original shape. For instance, bones will reabsorb or neoform, vessels will expand, muscles will become sore and tense; all with a view to restoring tensegrity, since the altered position or shape reestablishes it and rebalances the system.

A break in the tensegrity of a cell and the release of mediators inducing phenomena around it, with a view to reestablishing it, is known as mechanotransduction. In other words, mechanotransduction is a mechanism by which physical mechanical events are converted into biological cell and tissue events. It is by this means that the organism receives and absorbs mechanical stimuli and converts them into biological signs and events.

Surrounding bones, muscles, tendons and tissues form a complex system over which various types of force act. The chemical mediators released by the cells, as a result of force application, might cause mineralized tissue to reabsorb in some areas of the skeleton, while, at other sites, bone neoformation phenomena are induced. Therefore, tensegrity and mechanotransduction might Figuretively represent tools that daily sculpture the bones, a phenomenon also known as bone remodeling.

## Bones, periostea, muscles and primary bones, at the tooth socket and tendon attachments, form a system of forces

**Figure 1:**
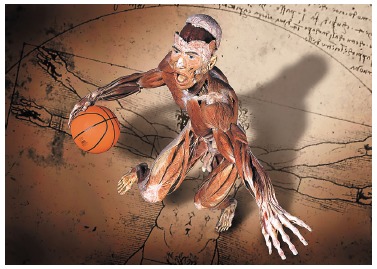
- Muscles and tendons apply loads over mineralized bone structures which, with a view to meeting functional demands, have thickness of trabecular and cortical bones increased while subject to stimuli that characterize the dynamics of bone remodeling.

Muscles and tendons are inserted into the bones in order to take us to the wonders of the world (Fig 1). The muscles are inserted into the bones by the periosteum, a thin layer of fibrous connective tissue connected to the cortical bone, thus forming its surface layers. The tendons have structural proteins directly fused to the bone, without interposition of the periosteum.

It is a very special bone also known as primary bone, which resembles that of an embryo. It is known as embryonic bone. Its composition and organization - more cellularized and less mineralized - render the primary bone more dynamic for renewal and functional adaptation. 

The bone seems to be wrapped up as a gift, since, at the surface, it is covered by a resistant protective membrane comprising the periosteum - a fibrous connective tissue made up of cellularized and vascularized layers. There are only two sites where the outer surface of bones is not covered by the periosteum. The first are the sites where tendons directly attach via primary bone; whereas the second one is the periodontal alveolar surface. 

At the tooth socket, the periodontal ligament acts as the periosteum and, similarly to tendons, it attaches directly to the bone by having structural proteins fused to the primary bone that surrounds it. Primary bone surrounding the tooth socket is also known as alveolar bone or bundle bone. Primary bone is always fused with structures that do not cease to transmit forces and require continuous and fast functional adaptation.

The bones also protect vital body parts, such as the brain, heart and lungs, in addition to containing bone marrow which produces the liquid that gives us life and protection: the blood. The bones, as the wonderful anatomical pieces that they are, perform a variety of functions, especially because they act together with a set of cartilage, tendons, ligaments and joints. 

## Bones have no angles or corners

In the human skeleton, as we observe the shape of bones, how they articulate or relate to the other parts of the body, we can see that there are no straight angles, corners, edges or pointy structures: everything is regular, smooth or round. Should bones not be round, they would wound soft tissues with which they relate at the periphery. Bone fractures or spurs are extremely painful due to having a pointy configuration. Bones must be round and smooth. 

Pointy edges or straight angles resulting from surgery at operated body cavities and sites will become round within a few weeks after surgery, since this is the natural and adaptive configuration of bones in relation to tendons, muscles and other soft tissues.

The bone crest will always have a stable round cervical end, and whenever it acquires a curve-like shape, the periosteum gradually disappears to allow the periodontal ligament tissue to continue. At the inner alveolar bone and periodontal surfaces, the periosteum is non-existent, with the periodontal ligament performing its functions at the site.

## Bone dynamism and its ability to adapt

Galileo, in 1638, was the first to suggest that bone shape is directly related to the forces applied to its structures. However, Julius Wolff, in 1892, was eloquent in proposing that the bone adapts or responds to forces.^3^

**Figure 2:**
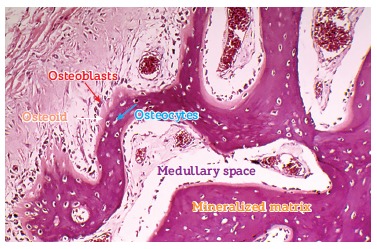
- Bone structures. The following are highlighted: trabeculae, a thin layer of osteoid, osteoblasts and especially osteoblasts, which constitute, via canaliculi and with a number of cytoplasmic extensions, a cell-to-cell network with mediators and trabecular as well as cortical bones, thus actively affecting bone remodeling (HE, 25X).

**Figure 3:**
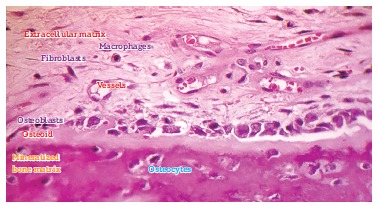
- On the surface, osteoblasts form new bone layers as they are stimulated by mediators released by local neighboring cells and which travel through the blood, or by osteocytes travelling through connected canaliculi (HE, 25X).

Athletes tend to have bigger, more voluminous and more mineralized bones because their structure and design are dynamically adapted according to function. Individuals with a sedentary lifestyle have thinner, less dense and less mineralized bones. At first, we learn that bones are dry, hard and resistant structures, as depicted by skeletons displayed at Departments of Anatomy and museums. However, they are in fact malleable, moldable, adaptable structures that seek to satisfy all functional demands we come up with. Bones are "moist" and have their surface composed of non-mineralized matrix thin layers (Figs 2 and 3). They are rich in water, minerals and a number of proteins and growth factors that most of times are hidden or associated with minerals.

The skeleton is thus an excellent partner, understanding and soft, which erases bruises and wounds as it remodels itself. The brain and the heart are different, as they are definite throughout life; they cannot regenerate and are left with permanent marks whenever injured. 

## THE BONE IS ABLE TO CONTROL ITS SHAPE

A young adult's skeleton undergoes complete renewal every 4-5 years and it is continuously transformed until the end of life: a 70-year-old man would have an average of seven complete bone renewals throughout life. 

**Figure 4:**
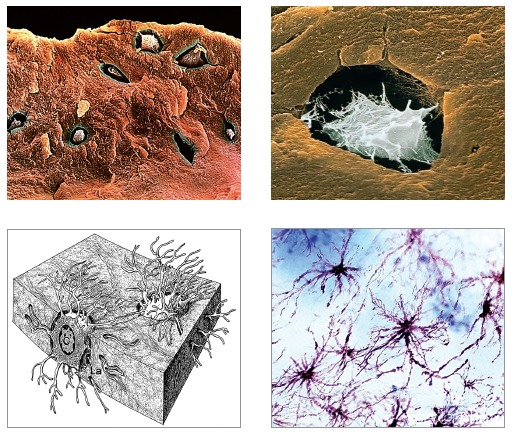
- Osteocytes and their typical shape, with dozens of extensions forming an effective network within the mineralized bone matrix. Lacunae where osteocytes are located are termed osteoplasts.

The mineralized structure of bone, both cortical and trabecular, has millions of lacunae as tiny as cells. Three-dimensionally, these lacunae have a spider-like shape with dozens of ramifications. They are empty, termed osteoplasts and their function is to enclose the osteocytes (Fig 4). 

**Figure 5:**
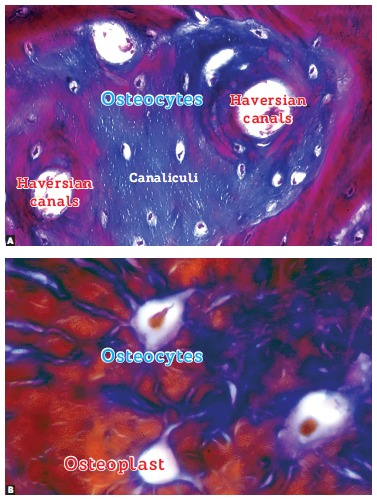
- Osteocytes within the mineralized matrix, revealing a number of cytoplasmic extensions inside the intercommunicating canaliculi (HE; A = 25X, B = 40X).

**Figure 6:**
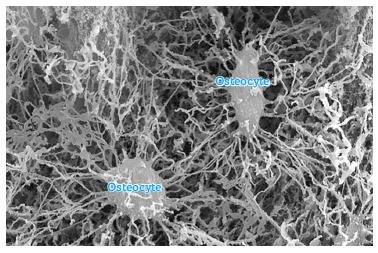
- Osteocytes were replaced by resin which, after polymerization, formed the shape of lacunae and canaliculi visible by scanning electron microscopy once the mineralized portion was completely removed by acids. As evinced, this network helps us comprehend how sensitive osteocytes in capturing bone deformation, albeit discreet.

Osteocytes have between 20 to 50 cytoplasmic extensions randomly distributed by means of tubules or bone canaliculi throughout the hard bone surface (Fig 5). Each one of these cells interconnects with other 40 to 50 cells. Imagine several spiders holding hands or making cell-to-cell contact with other 20 to 30 spiders (Fig 6). 

Osteocytes form an interconnecting network within the mineralized bone structure. Whenever forces are applied during movement, deformation is established as a result of stretching or compression, thus allowing the network to apprehend the changes in shape while immediately communicating or transferring the new situation to the network of osteocytes.

In addition to cell-to-cell contact, one of the most effective ways of passing on a message is to release substances, products or chemical mediators to the inner or outer surfaces of cells with, for instance, the following biochemically codified information: 

... change your current shape, we need or are required to adapt to the new situation, improve your design. Reabsorb at compression sites, deposit at sites of stretching.

Every day, bone design adapts to function. A clear demonstration of flexibility, adaptability and will to serve the whole. The ideal, absolute supremacy of the whole over the parts. 

## THE COMPONENTS

**Figure 7:**
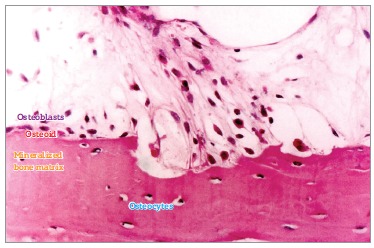
- Flow of mononuclear cells towards the bone surface exposed by local displacement of osteoblasts, resulting from the action of mediators and changes in local conditions, such as pH reduction due to cellular stress. Note discreet Howship lacunae, with special attention to osteocytes within the mineralized bone matrix (HE, 25X).

**Figure 8:**
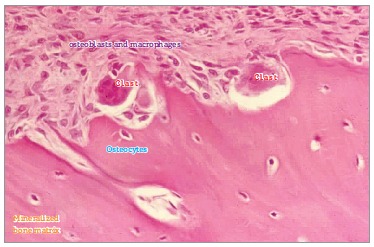
- Two bone modeling units (BMUs) in Howship lacunae, with special attention to osteocytes, osteoblasts and macrophages as important components.

**Figure 9:**
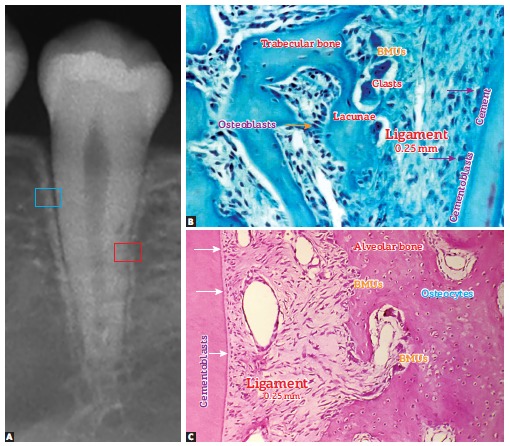
- On periodontal ligament bone surface, bone modeling units (BMUs), as it occurs in the entire body, continuously renew the bone structures, thus causing minimal natural tooth movement throughout one's life. Cementoblasts on root surface, without membrane receptors for bone mediators, do not take part in bone remodeling (B = Mallory, 25X, C = HE, 25X).

Bone surface cells act as workmen: osteoclasts - or simply clasts - pull walls down or undo overlays (Figs 7 and 8); osteoblasts build up and reinforce columns and other structures (Figs 3 and 9). Bone surface cells are controlled by osteocytes; however, not too long ago, researchers believed they were cells isolated at the farthest of mineralized matrix: it was a complete mistake!

Should one's desire be to reinforce the skeleton, it is necessary to deform and stretch muscles and tendons, so as to deform the bone which has a deflection capacity; thus stimulating the network of osteocytes to adapt to the design desired by whom planned the functional demand! 

Should osteocytes remain at constant rest or in homeostasis, they have no stimuli to renew the skeleton and adapt it to one's lifestyle. Bones become increasingly fragile, less hard and little resistant! The skeleton is an ongoing metamorphosis - a process also known as bone remodeling or bone turnover. While in children and adolescents the bones are modeled and remodeled at the same time in order to satisfy development and growth, adults undergo ongoing bone remodeling.

An adult skeleton has between 1 and 2 million microscopic bone resorption sites. At every site, bone remodeling occurs in four phases, as follows.

## **Activation:** precursor cells respond to physical and hormonal signs within an area of bone surface where they organize as clasts.

## **Resorption:** after 7 to 15 days, activated clasts open up small, shallow cavities, or 60-µm Howship lacunae.

**Reversion:** once resorption is complete, clasts activity ceases by means of removal of stimuli. Within 7 to 14 days, a new thin line of bone matrix is deposited, with collagen fibers, contrasting with the well-organized, lamellar collagen surface. This line sets the limit of resorption that ceases with neoformation of bone.

**Formation:** pre-osteoblasts that have been recently required begin to deposit the new bone matrix. The time required between deposit of immature non-mineralized bone and mature bone is 21 days. In general, the time required for filling and return to normality is from 8 to 12 weeks.

## WHY IS IT SO?

For the organism to have a balanced organization and functioning at varied orders of magnitude, *calcium ion is key to life:* it is part, directly or indirectly, of nearly all biological processes inherent to human life. Generally speaking, 99% of calcium is found in the bones, whereas only 1% is used for overall metabolism. In one's organism, calcium is associated with proteins from which it easily disconnects whenever induced or necessary.

The availability of calcium to cells and tissues must be ensured, since it is related to maintenance of life. Between meals, the serum levels of calcium mainly come from the skeleton acting as an active mineral reservoir. Matrix and minerals apposition - by osteoblasts and osteocytes - as well as bone resorption for disassembly of focal adhesion and ions release into the blood by clasts primarily aim at reaching maintenance of serum levels of ions, particularly calcium.

**Figure 10:**
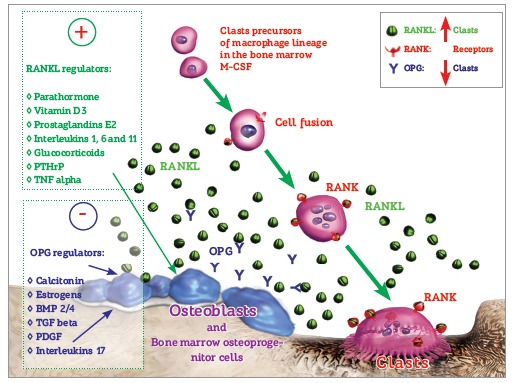
- At the bone environment, there are mediators intrinsic to bone modeling units. They speed up or inhibit bone remodeling according to stimuli extrinsic to these units, and are represented by bone remodeling local and systemic mediators. A few mediators stimulate osteoblasts, osteocytes and other local cells to release RANKL which, in turn, stimulates clastic activity. Other mediators, however, stimulate the production of osteoprotegerin, or OPG, which reduces the effect of RANKL by connecting to the molecules and preventing them to interact with clastic membrane receptors or RANKL.

**Figure 11:**
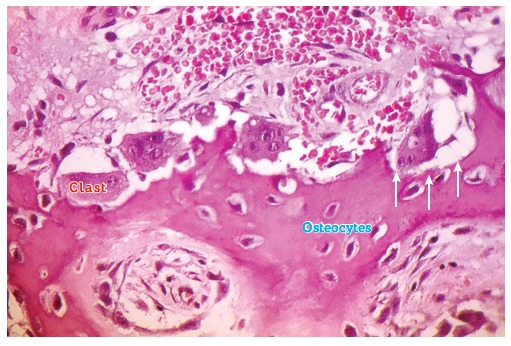
- Various bone modeling units and their clasts in their respective Howship lacunae, and a network of osteocytes associated with the resorptive phenomenon, potentially releasing mediators and directly affecting local bone remodeling (HE, 25X).

The cells of the **four parathyroid glands** (positioned on the side of the thyroid, in the trachea) capture the reduction in serum levels of calcium and release **parathormone** directly into the blood, the main mediator inducing clastic activity. Once parathormone has been distributed and begins to rapidly interact with the other body cells via membrane receptors - especially osteoblasts that control the clasts - it establishes resorptive activities for ions release from the bones and return to normality of the serum levels (Figs 10 and 11).

**Vitamin D3** - which molecularly is not considered a vitamin, but a hormone - plays a major role in this process, as it increases calcium absorption in the intestinal mucosa while also being an important stimulator of osteoclastogenesis, or the maturation of new clasts.

Within a few minutes or hours, this resorptive activity might increase significantly the serum level of calcium, which is detected by C cells, also called parafollicular cells in the thyroid, producers of **calcitonin.** Calcitonin release into the blood inhibits bone resorption in other skeletal cells, thus contributing to the prevalence of bone apposition phenomena while distributing mineral ions throughout the bone matrix. **Estrogens** also contribute to this reversion from resorption to predominant bone apposition (Figs 10 and 11).

This dynamics of continuous apposition and resorption, for maintenance of ion balance in the body, allows the bone to adapt significantly to a number of daily functional demands. These are the reasons why an analysis of bone structure and composition allows one to determine the modern individual lifestyle and skeletal as well as muscular functional patterns, as the bone adapts and renews itself at every stage of life! 

## LOCAL BONE REMODELING: HOW DOES IT OCCUR?

In specific areas, adaptation of bone shape and volume are rendered necessary and occur regularly. Whenever forces are applied to a specific area, osteoblasts, osteocytes and other cells, such as vascular endothelial cells, at blood vessel walls, and neural threads as well as neighboring connective tissue fibroblasts cells, will locally deform. 

Cell deformation affects the spatial configuration of the cytoskeletons responsible for the original shape of each cell. In general, the shape of cells is stable, steady and typical of each one of the 206 types of cells found in the human body. Cell deformation represents a break in tensegrity: the cell is subject to stress, in other words, cellular metabolism increases and so does the amount of mediators released to the outer surface of cells where extracellular matrix is located. Cytokines and growth factors induce local phenomena in order to have tensegrity restored or to create new tensegrity or local balance. These phenomena include local bone resorption or neoformation, provided that the environment where deformation occurs be central or peripheral bone tissue.

In short, it is reasonable to claim that bone remodeling occurs as a whole; however, at specific areas, if necessary, specific remodeling might occur, particularly if the number of local mediators, such as cytokines and growth factors, increase significantly within a specific region. Orthodontic and/or orthopedic movement is a good example of therapeutically intended induced local bone remodeling.

## ANCHORAGE: LIMITS AND POSSIBILITIES

In the maxilla and the mandible (Fig 9), as in any other bone, processes and general design, including volume, shape and other minor details, are determined by functional demand. The alveolar process gives support to teeth without which there is no reason that justifies the existence of this bone protuberance. In edentulous patients, there is a tendency towards atrophy, causing the alveolar process to disappear completely. 

The **use of osseointegrated implants** soon after tooth loss contributes at least to reduce alveolar bone loss. Masticatory load over osseointegrated implants is transmitted to neighboring bone tissues; thus, the structure tends to remain unchanged, since there is continuous functional demand.

**Teeth move naturally throughout life** and follow the vectors initially provided by modeling and then remodeling at adulthood. The balance of muscle and soft tissue forces acting over teeth added to masticatory load associated with alveolar bone support determine more or less stability of teeth into the dental arch. Dental crowding is considered an imbalance of this system and it is found, in most people, as some sort of oral aging.

Initially, **induced tooth movement** guides natural movement and, many times, induces forces intentionally directed towards speeding up bone remodeling in one of the root surfaces, so that a new position of teeth is achieved. 

On the compression side, periodontal ligament cells release several mediators of cellular stress, thus stimulating bone cells to rapidly reabsorb on the periodontal surface of the alveolar bone (Figs 13 and14). In areas where the periodontal ligament is stretched out, bone apposition phenomena are predominant, since mediators of cellular stress are found in smaller amounts, thus inducing apposition phenomena.

**Figure 12:**
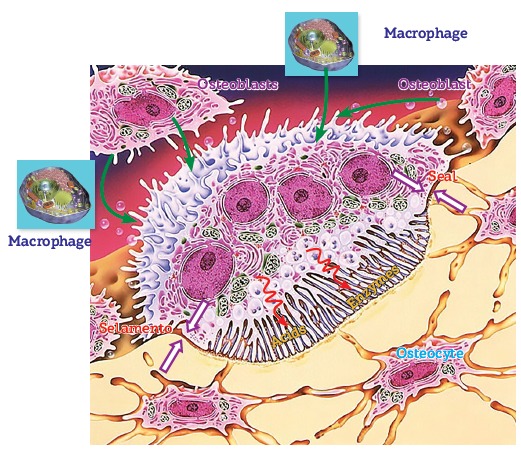
- Diagram illustrating the function and organization of a bone modeling unit. Each BMU is controlled by osteoblasts and secondarily by macrophages via RANKL mediators. At the active or brush border, acids and enzymes are released by an effective sealing zone formed by molecular fusion between membrane and bone proteins. Note the relationship established with osteocytes.

**Figure 13:**
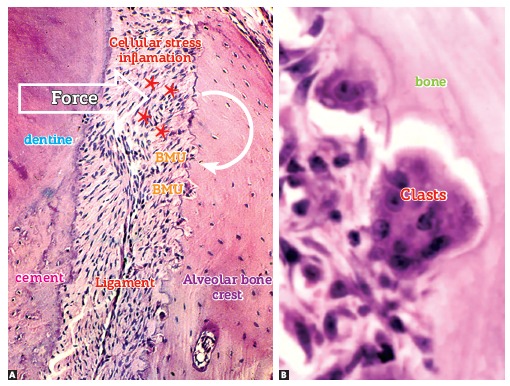
- The bone crest periodontal surface of a tooth subject to induced tooth movement reveals areas without osteoblasts, in addition to stress and inflammation of ligament (asterisks) at the periphery. Subsequently, bone modeling units, or BMUs, settle down and begin the process of bone resorption. In B, note the BMU components in function, with clasts and mononuclear cells located at the periphery while representing osteoblasts and macrophages (A and B = HE, 10 and 40X).

**Figure 14:**
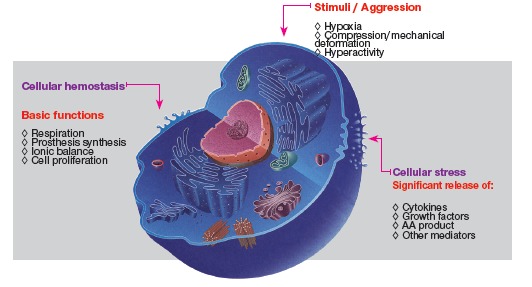
- Stimuli or aggressive agents frequently found in the bone and teeth subject to force application. They are responsible for releasing mediators key to the biological process of bone remodeling.

**Induced tooth movement** is a therapeutically and esthetically intended biological phenomenon; however, it **has its limitations**. These limitations are associated not only with the speed in which movement occurs in time, but also with the harmonious extent of movement involving the whole set of teeth.*For patients with time and extent restraints for induced tooth movement, orthodontists usually opt for orthognathic surgery.*

Due to absence of periodontal ligament and natural tooth movement, osseointegrated implants present with more stability. Including osseointegrated implants as anchorage points in rehabilitation treatment planning performed with mechanics associated with miniplates is a perfectly feasible option, particularly from a biological and physical perspective. Anchorage forces, albeit substantial, are much less significant than those forces produced by daily mastication.

## ANCHORAGE: boosting possibilities and the role of Orthodontics in oral rehabilitation

One of the factors responsible for increasing orthodontic treatment time and reducing the potential for certain types of tooth movement extension was lack of anchorage. Initially, mini-implants were introduced with promises of absolute anchorage, but later on they presented with limitations on supporting heavy loads, in other words, anchorage proved to be not as absolute. They aid in the resolution of many cases, most of them requiring additional anchorage beyond that offered by teeth; however, they also have their limitations.

A few cases of extensive movement, thought to be impossible for many decades, required significant reshaping of the maxilla and mandible. There remained cases which, despite anchorage offered by mini-implants, could not be solved. In some cases, orthognathic surgery itself was not the ideal solution, under any circumstances. 

More complex cases, particularly in terms of esthetics and function, require a combination of Orthodontics with other clinical specialties for patient's complete rehabilitation. Nevertheless, these cases present with high risks of yielding unsatisfactory outcomes. The limitations of results are usually attributed to technical, physiological and anatomical reasons, such as impaired mechanical possibilities due to lack of anchorage.

The use of miniplates is a great opportunity for anchorage and offers new solutions of maxillary orthodontic as well as orthopedic mechanics, as it allows force to act with greater intensity and to a greater extent, reaching almost the entire extension of the maxilla and mandible. 

*Under no circumstances, the orthodontic and orthopedic use of miniplates replaces or imply changing orthognathic surgery treatment planning.* It solely implies that the possibilities of success in more complex cases, in which all therapeutic actors must be used in synergy, are improved. Miniplates are not indicated and do not solve all cases, which also applies to orthognathic surgery. 

The sculptor, such as the surgeon, uses the marble or wood before him to make sculptures as planned, making his art real based on what had been envisioned! Moving teeth and changing the shape of bones on the basis of anchorage and active forces, thanks to the dynamics of bone remodeling, might be compared to the art of a ceramic sculptor, the artist of clay (Fig 18). Taking advantage of a malleable material, fingers, hands and tools - such as forces exerted by bones - the artist creates new shapes and produces new details, so as to achieve the final design. There are two ways by means of which final outcomes can be achieved: the beauty and function of art as part of human imagination. **To the surgeon, the art of carving; to the orthodontist, the art of modeling: beauty shall benefit human beings!**

**Figure 15:**
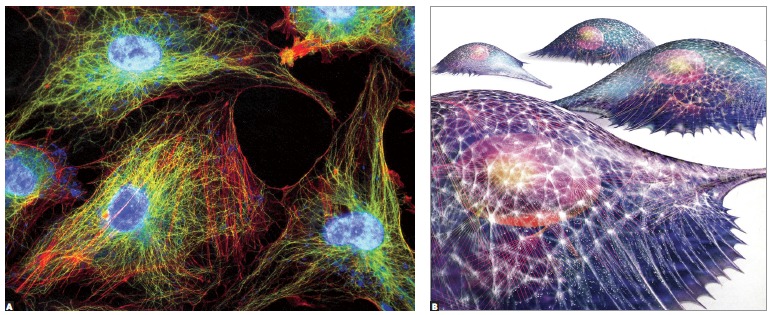
- Cells with cytoskeleton proteins evinced by immunofluorescence, and diagram in B. Proteins, in red and green, reveal the structure that maintains cell shape and which provides the cell with mobility, whenever necessary. Cytoskeleton proteins are connected with integrins within the cell membrane and with the nuclear membrane at the center (in blue).

**Figure 16:**
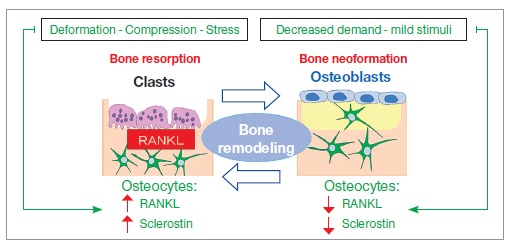
- Diagram illustrating how osteocytes actively participate in bone remodeling as mechanotransductors. Deformation of the network of osteocytes induces mechanical stress. RANKL local levels are raised, with a higher number of active clasts and greater release of sclerostin by osteocytes. Once the response to stimulus is adequate, there is a reduction in the number and activity of clasts, and a reduction in sclerostin levels by osteocytes.

**Figure 17:**
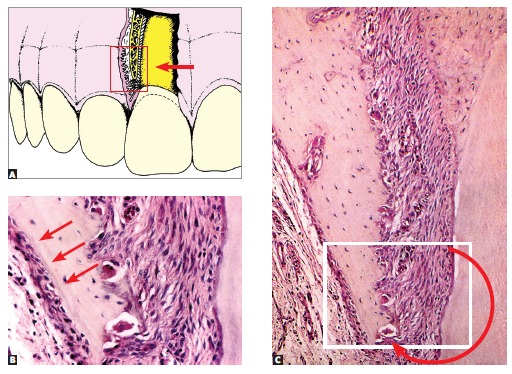
- The periosteum, within the area corresponding to induced tooth movement (arrow in B), receives mechanical stimuli also provided by mediators released by osteocytes subject to deformation and acting as mechanotransductors by reacting with deposition of new bone layers on the cortical surface (arrows in C), thus changing the shape, volume and size of the jaws, in addition to affecting teeth positioning (HE; 25X).

**Figure 18:**
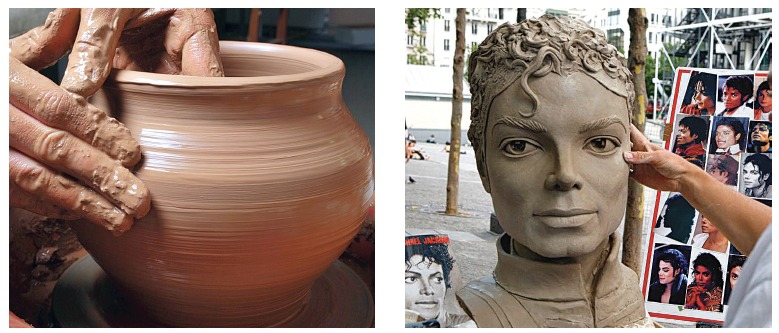
- Clay modeling is performed with force application intended to adapt the material to the design planned by the artist. It is analogy to the application of forces by means of mini-implants and miniplates on the tridimensional network of osteocytes which will cause the bone structure to adapt, thus determining a new shape and tensegrity for the modified structure.

Understanding the function of miniplates in orthodontic and orthopedic treatment planning requires mastering the concepts of bone biology within the context of bone remodeling. Drawing analogies might render understanding faster and applicable.

## MINIPLATES AND MINI-IMPLANTS "ACT" OVER OSTEOCYTES!

The bone is composed of more than 50% of organic matter, including water. This suggests that the bone is highly malleable and deforms when subject to compression or stretching. The intercellular network of osteocytes is immediately altered (Figs 4 to 6), thus affecting the tridimensional pattern of each osteocyte cytoskeleton (Fig 15). 

The inner shape of osteocytes is determined by another network that resembles the tridimensional network of osteocytes itself, but formed by interconnected proteins. This network is known as cytoskeleton (Fig 15) in which loss of balance in shape stability implies cellular stress characterized by an increase in metabolism and secretion of chemical mediators which will induce a number of phenomena, regardless of which, with a view to restoring physical and metabolic stability (Fig 14).

In other words, bone deformation increases metabolism and message exchange among osteocytes by means of mediators release, as well as among osteocytes, osteoblasts and clasts on trabecular and cortical bone surfaces (Figs 11, 12 and 13).

Bone deformation caused by compression and stress raises the production of RANKL mediators and osteocyte-derived sclerostin, while also enhancing the mechanisms of resorption by clasts (Fig 16). On the other hand, reduced functional demand, under mild stimuli, leads to a decrease in the release of these mediators by osteocytes, thus causing bone neoformation phenomena by osteoblasts to predominate (Fig 16). 

Osteocytes biochemically control bone cell activity on structural surfaces, signaling where resorption and apposition of bone layers should take place in order to adapt bone shape, design or anatomical profile to the functional demand that represents the deformity (Fig 17). 

The role osteocytes play in bone biology was practically ignored for decades. Analysis of leading-edge studies on bone homeostasis reveals that the role played by osteocytes is key to understanding metabolical bone diseases and bone remodeling and repair processes. *Osteocytes account for 90 to 95% of bone cells.*

The forces applied at **induced tooth movement** minimally deform the teeth; instead, they deform - to a greater extent - the alveolar processes externally (Fig 17) as well as the basal bones of the jaws. *Orthodontic movement does not affect the inner part of the tooth socket only, but it also affects the size, thickness as well as shape of trabeculae found in neighboring bones and the outer surface of their cortical bones, thus establishing or changing itself into layers of bone juxtaposed by the underlying periosteum.* On the basis of osteoplasts, osteocytes, acting as architects, control all the workers juxtaposed on inner and outer bone surfaces (Fig 17).

The **use of osseointegrated implants** does not only imply in a still and inactive abutment screwed to the bone structure, only. Its function, performed via loading, affects the surrounding areas and, albeit afar, the neighboring trabecular bone and its underlying cortical bone. Continuous remodeling takes place directly - and on a daily basis - in areas surrounding it, and the bone is readjusted every minute to all necessary and functional demands.

**Orthopedic appliances,** with their plates, shields and other devices, back up changes based on the following grounds of bone pathophysiology understanding: the tridimensional network of osteocytes that controls bone shape. Changes in this network of osteocytes implies in changes in bone shape and repositioning of structures involved, namely: teeth, muscles and tendons. The dynamism offered by bone remodeling implies in continuous adjustment of the body to functional demands.

The **use of mini-implants** increased the potential for appliance treatment planning towards a more effective biomechanics in tooth repositioning, with outstanding outcomes usually restricted to a small area of the maxilla where they could be used as anchorage sites.

**Figure 19:**
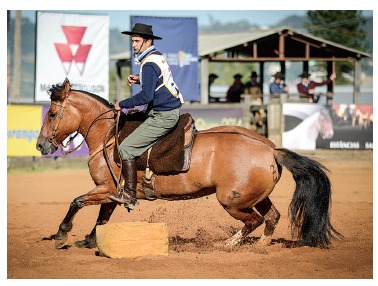
- Tooth movement performed by means of conventional orthodontic appliance, regardless of the type of bracket, might be compared to forces transmitted by the reins placed on the head of a horse controlled by a horseman who directly affects command of what is ahead of him. The head and the body immediately move around him. The horseman on the horse might be compared to the bracket bonded to the anchorage tooth.

**Tooth movement** performed by means of conventional **orthodontic appliance,** regardless of the type of bracket, might be compared to forces transmitted by the reins placed on the head of a horse controlled by a horseman who directly affects command of what is ahead of him. The head and the body immediately move around him. The horseman on the horse might be compared to the bracket bonded to the anchorage tooth (Fig 19).

The **use of mini-implants** might be compared to a cart or a small carriage where the coachman, with the reins placed on the animal or a couple of animals side-by-side, controls the forces applied to the animals' mouths, restraining them and inducing right, left or forward movements. The reins would be the wires; whereas the devices in the animal's mouth would be the brackets. The coachman might be compared to the mini-implant. Thus, the cart changes and operates according to the forces applied and the needs of the coachman. 

**Figure 20:**
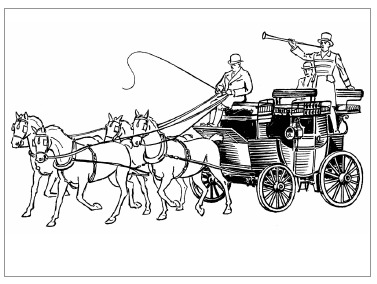
- Analogy: Miniplates correspond to larger carriages, with various animals. The reins or wires must be well calibrated and secured, the coachman must be properly and the carriage must be stable. Miniplates can control, albeit afar, the shape of the network of osteocytes in the bone that gives support to anterior incisors and canines, in addition to influencing midline position and relationship.

**Miniplates** correspond to larger carriages, with two to three pairs of animals, used for grandiose events and journeys at old English imperial times (Fig 20). The reins - or wires - must be well calibrated and secured to the animals' mouth. The coachman must be properly trained to excellence, and the carriage must be stable enough to ensure passengers' safety throughout the desired path. The coachman knows exactly what to expect from each couple of animals. The first, on the front, leads the others in harmony by the forces exerted by the reins placed on the beautiful carriage.

**Figure 21:**
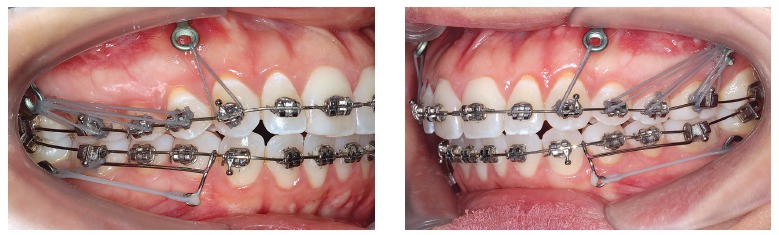
- Miniplates offer anchorage to a number of elastics and devices, which allows the shape of the maxilla and mandible to be remodeled and have their relationship with all other anatomical structures to be changed. This example of miniplates clinical use allows the analogy with reins to be applied. Clinical case granted by Dr. Ertty Silva.

**Figure 22:**
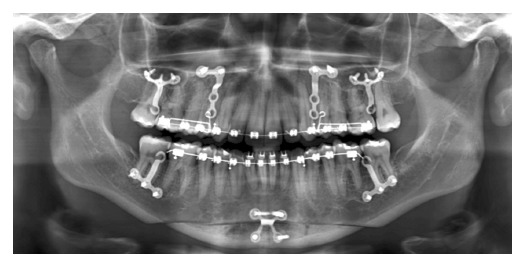
- Miniplates provide greater anchorage, as they are secured to a wider base with two to three screws fixed in thicker bone areas where ordinary bone remodeling tends to occurs slowly when compared to other areas, as depicted by the images. Clinical case granted by Dr. Ertty Silva.

Similarly to a majestic carriage, miniplates can control, albeit afar, the shape of the network of osteocytes in the bone that gives support to anterior incisors and canines. They can, therefore, affect the position and midline relationship between the maxilla and the mandible. 

In other words: anchorage offered by miniplates allows force to be exerted so intensely, so as to be transmitted further. This property enhances orthodontic, orthopedic and rehabilitation treatment outcomes with implications for esthetics and function that are restored to the patient. Miniplates might allow a broader remodeling of the maxilla and mandible than the limited remodeling resulting from the use of brackets and mini-implants. 

## CARE AND LIMITS ON THE USE OF MINIPLATES AND MINI-IMPLANTS

**Area contamination.** A possible drawback of miniplates is the continuous communication between the inner environment and the oral environment by means of the miniplate external loop. Patient's guidance/awareness about oral hygiene, in addition to technical care during miniplate placement, is key to avoid this type of problem. Patients must be informed on a number of oral antisepsis protocols to be performed while the miniplates are in function.

Microbial biofilm and bacteria build-up on miniplates might trigger inflammatory processes with exudate formation and the potential need for miniplate removal. Nevertheless, it is worth noting that patient's compliance and awareness is compulsory when deciding whether to use miniplates or not. 

Importantly, due to the opening offered by miniplates, patients must be healthy, since uncontrolled systemic diseases, such as uncontrolled diabetes mellitus, long-term use of corticosteroids, immunosuppression and anemia, might cause the area to be contaminated more often.

**Unthread screws and/or miniplate.** Incidents that might occur with all procedures. The chances are reduced by properly selecting the area of insertion, technical elements and the technique to be used. 

**Sinus problems.** Should the maxillary/sinus cortical bone be perforated by fixation screws faced towards the maxillary sinus, chronic as well as local maxillary sinusitis might occur, especially considering the length and diameter of screws. 

In general, cases of sinus diseases are subclinical and asymptomatic, tending to disappear within a few days. However, the following might originate at these sites: 1) sinus polyps represented by isolated areas of swelling in the sinus mucosa; or 2) cyst of maxillary sinus mucosa represented by isolated lumps filled with mucus at the submucosa. Both cases, sinus polyps and cyst of maxillary sinus mucosa, are asymptomatic and require no treatment, since they disappear completely within a few weeks or days without further consequences.

**Tooth roots perforations or gliding.** Tooth roots are occasionally accidently damaged. In cases of mini-implant gliding, removal implies root surface repair within a few weeks, with cement neoformation and reinsertion of periodontal collagen fibers. 

Root perforation caused by previous drilling requires pulp vitality to be assessed because, in the event of pulp space damage, aseptic pulp necrosis might be achieved, with the need for endodontic treatment. Should perforation not have reached the pulp space, mini-implant removal implies repair with cement and reinsertion of periodontal collagen fibers within a few weeks or months. Normality will be restored without the need for endodontic treatment.

## FINAL CONSIDERATIONS

Osteocytes form a tridimensional network that controls bone design, coordinating cell activity on trabecular and cortical bone surfaces, especially osteoblasts and clasts. 

Miniplates and mini-implants are used for support or anchorage, so as to allow all other orthodontic and orthopedic components, albeit afar, to deform and stimulate the network of osteocytes to command bone design remodeling on "functional demand" created by force and its vectors. In the transmission of forces, whether near or distant, based on anchorage offered by miniplates, it is possible to change the position, shape, size and relationship established between the bones of the jaws. 

There is a potential for accidents and incidents in all technical procedures; however, safety and prevention reduce their frequency, which does not refrain these procedures from being employed. We must understand bone biology and continuous skeletal remodeling in order to perform patient's safe and accurate rehabilitation treatment while raising the possibilities of intervention with a view to restoring patient's esthetics and function.

## References

[1] Bakker AD, Soejima K, Klein-Nulend J, Burger EH (2001). The production of nitric oxide and prostaglandin E(2) by primary bone
cells is shear stress dependent. J Biomech.

[2] Baron R, Hesse E (2012). Update on bone anabolics in osteoporosis treatment: rationale, current
status, and perspectives. J Clin Endocrinol Metab.

[3] Bonewald, Lynda F (2006). Mechanosensation and transduction in osteocytes. Bonekey Osteovision.

[4] Bonewald LF (2006). Osteocytes as multifunctional cells. J Musculoskelet Neuronal Interact.

[5] Bonewald LF (2011). The amazing osteocyte. J Bone Miner Res.

[6] Burr DB, Robling AG, Turner CH (2002). Effects of biomechanical stress on bones in animals. Bone.

[7] Crockett JC, Rogers MJ, Coxon FP, Hocking LJ, Helfrich MH (2011). Bone remodelling at a glance. J Cell Sci.

[8] Ehrlich PJ, Noble BS, Jessop HL, Stevens HY, Mosley JR, Lanyon LE (2002). The effect of in vivo mechanical loading on estrogen receptor alpha
expression in rat ulnar osteocytes. J Bone Miner Res.

[9] Feng JQ, Ward LM, Liu S, Lu Y, Xie Y, Yuan B (2006). Loss of DMP1 causes rickets and osteomalacia and identifies a role for
osteocytes in mineral metabolism. Nat Genet.

[10] Kamioka H, Honjo T, Takano-Yamamoto T (2001). A three-dimensional distribution of osteocyte processes revealed by
the combination of confocal laser scanning microscopy and differential
interference contrast microscopy. Bone.

[11] Krstic RV (1994). Human microscopic anatomy.

[12] Lane NE, Yao W, Balooch M, Nalla RK, Balooch G, Habelitz S (2006). Glucocorticoid-treated mice have localized changes in trabecular bone
material properties and osteocyte lacunar size that are not observed in
placebo-treated or estrogen-deficiente mice. J Bone Miner Res.

[13] Lanyon LE (1993). Osteocytes, strain detection, bone modeling and
remodeling. Calcif Tissue Int.

[14] Nakashima T, Hayashi M, Fukunaga T, Kurata K, Oh-Hora M, Feng JQ (2011). Evidence for osteocyte regulation of bone homeostasis through RANKL
expression. Nat Med.

[15] Parfitt AM (1977). The cellular basis of bone turnover and bone loss: a rebuttal of the
osteocytic resorption--bone flow theory. Clin Orthop Relat Res.

[16] Poole KE, van Bezooijen RL, Loveridge N, Hamersma H, Papapoulos SE, Löwik CW (2005). Sclerostin is a delayed secreted product of osteocytes that inhibits
bone formation. FASEB J.

[17] Raab-Cullen DM, Thiede AM, Petersen DN, Kimmel DB, Recker RR (1994). Mechanical loading stimulates rapid changes in periosteal gene
expression. Calcif Tissue Int.

[18] Skerry TM, Bitensky L, Chayen J, Lanyon LE (1989). Early strain-related changes in enzyme activity in osteocytes
following bone loading in vivo. J Bone Miner Res.

